# Absence of Type I Interferon Autoantibodies or Significant Interferon Signature Alterations in Adults With Post–COVID-19 Syndrome

**DOI:** 10.1093/ofid/ofad641

**Published:** 2023-12-19

**Authors:** Martin Achleitner, Nina K Mair, Juliane Dänhardt, Romina Kardashi, Milo A Puhan, Irene A Abela, Nicole Toepfner, Katja de With, Waldemar Kanczkowski, Natalia Jarzebska, Roman N Rodionov, Christine Wolf, Min Ae Lee-Kirsch, Charlotte Steenblock, Benjamin G Hale, Stefan R Bornstein

**Affiliations:** Department of Internal Medicine III, University Hospital Carl Gustav Carus, Technische Universität Dresden, Dresden, Germany; Institute of Medical Virology, University of Zurich, Zurich, Switzerland; Life Science Zurich Graduate School, ETH and University of Zurich, Zurich, Switzerland; Department of Internal Medicine III, University Hospital Carl Gustav Carus, Technische Universität Dresden, Dresden, Germany; Department of Internal Medicine III, University Hospital Carl Gustav Carus, Technische Universität Dresden, Dresden, Germany; Epidemiology, Biostatistics and Prevention Institute, University of Zurich, Zurich, Switzerland; Institute of Medical Virology, University of Zurich, Zurich, Switzerland; Department of Infectious Diseases and Hospital Epidemiology, University Hospital Zurich, Zurich, Switzerland; Department of Pediatrics, University Hospital Carl Gustav Carus, Technische Universität Dresden, Dresden, Germany; Division of Infectious Diseases, University Hospital Carl Gustav Carus, Technische Universität Dresden, Dresden, Germany; Department of Internal Medicine III, University Hospital Carl Gustav Carus, Technische Universität Dresden, Dresden, Germany; Department of Internal Medicine III, University Hospital Carl Gustav Carus, Technische Universität Dresden, Dresden, Germany; Department of Internal Medicine III, University Hospital Carl Gustav Carus, Technische Universität Dresden, Dresden, Germany; Department of Pediatrics, University Hospital Carl Gustav Carus, Technische Universität Dresden, Dresden, Germany; Department of Pediatrics, University Hospital Carl Gustav Carus, Technische Universität Dresden, Dresden, Germany; Department of Internal Medicine III, University Hospital Carl Gustav Carus, Technische Universität Dresden, Dresden, Germany; Institute of Medical Virology, University of Zurich, Zurich, Switzerland; Department of Internal Medicine III, University Hospital Carl Gustav Carus, Technische Universität Dresden, Dresden, Germany; School of Cardiovascular and Metabolic Medicine and Sciences, Faculty of Life Sciences and Medicine, King's College London, London, UK; Department of Endocrinology, Diabetology and Clinical Nutrition, University Hospital Zurich and University of Zurich, Zurich, Switzerland

**Keywords:** autoantibodies, COVID-19, interferon, post–COVID-19 syndrome

## Abstract

Genetic defects in the interferon (IFN) system or neutralizing autoantibodies against type I IFNs contribute to severe COVID-19. Such autoantibodies were proposed to affect post–COVID-19 syndrome (PCS), possibly causing persistent fatigue for >12 weeks after confirmed SARS-CoV-2 infection. In the current study, we investigated 128 patients with PCS, 21 survivors of severe COVID-19, and 38 individuals who were asymptomatic. We checked for autoantibodies against IFN-α, IFN-β, and IFN-ω. Few patients with PCS had autoantibodies against IFNs but with no neutralizing activity, indicating a limited role of type I IFNs in PCS pathogenesis. In a subset consisting of 28 patients with PCS, we evaluated IFN-stimulated gene activity and showed that it did not correlate with fatigue. In conclusion, impairment of the type I IFN system is unlikely responsible for adult PCS.

Type I interferons (IFNs) are essential to limit severe viral disease; thus, dysfunction of the IFN system can be associated with serious life-threatening infections [1]. In the context of the recent SARS-CoV-2 pandemic, genetic defects in the IFN system or the presence of neutralizing type I IFN autoantibodies in patients who are infected has been associated with severe disease [2–5]. Specifically, neutralizing autoantibodies against IFN-α and/or IFN-ω have been detected in at least 10% of patients critically ill with COVID-19 pneumonia but are rarely found in patients with asymptomatic or mild disease [3]. As such, disruption of the IFN system through autoantibodies against type I IFNs appears to contribute to severe illness and death in a significant proportion of patients with COVID-19 [6, 7].

While the majority of patients with COVID-19 recover completely without sequelae, about 20% continue to show symptoms or even develop new symptoms 12 weeks after initial diagnosis, collectively known as post–COVID-19 syndrome (PCS) [8]. PCS is characterized by a variety of symptoms, including fatigue, headaches, sleep problems, dyspnea, exertion intolerance, depression, or anxiety [8]. The pathogenic mechanisms underlying PCS are only partly understood, but viral persistence [9, 10], endocrine dysregulation [11, 12], ongoing autoimmune responses [13], and persistent inflammation [14] have been suspected to play a role.

Little is known, however, about the potential involvement of dysregulated type I IFN signaling in the pathogenesis of PCS. Autoantibodies against type I IFNs, as well as persistent activation of the IFN system, were recently proposed to contribute to the pathogenesis of PCS, at least in a subset of patients with pulmonary manifestations [15, 16]. In particular, it was speculated that SARS-CoV-2–associated activation of IFN signaling might promote the generation of autoantibodies against type I IFNs [15] and enhance local inflammation [16, 17] potentially driving PCS symptoms. A limited number of studies have analyzed the presence of such autoantibodies in patients with PCS. In one such study, only autoantibodies against IFN-α2 were screened for, and their detection in 2 of 121 patients with PCS [18] suggests a limited role in the pathogenesis of this syndrome. It has also been suggested that an elevated IFN-stimulated gene (ISG) signature might play a role in the pathogenesis of chronic fatigue. This is notable, considering that an activated ISG signature and fatigue are hallmarks of several autoimmune diseases [19, 20], and it is suggested that ongoing activation of the IFN system may contribute to chronic fatigue [21–23]. However, to our knowledge, such links have yet to be investigated comprehensively in patients with PCS.

In this study, we therefore sought to address the question of whether autoantibodies against type I IFNs (IFN-α, IFN-β, and IFN-ω) or upregulation of IFN-stimulated genes is associated with the presence of persistent fatigue after infection with SARS-CoV-2.

## METHODS

### Ethics

All participants provided written informed consent.

### Study Cohort and PCS Assessment

This study included 128 adult patients from the long COVID-19 outpatient clinic at the University Hospital in Dresden, Germany, and employees who reported fatigue. These patients tested positive for SARS-CoV-2 and reported having fatigue symptoms for at least 12 weeks after initial diagnosis (PCS group).

A standardized questionnaire (FACIT-F; https://www.facit.org/measures/FACIT-F) approved for the assessment of fatigue in autoimmune diseases [24] was used to measure the degree of fatigue. Moreover, a visual analog scale was used to determine levels of tiredness. A control group consisted of 38 patients (employees of the University Hospital in Dresden) who developed only mild COVID-19 and demonstrated no signs of fatigue. Furthermore, the study included a group of 21 patients with severe COVID-19 (ie, hospitalized because of COVID-19; Table 1).

**Table 1.** Patient Characteristics

	Control (n = 38)	PCS (n = 128)	Severe COVID-19 (n = 21)
Female, %	81.6	77.3	14.3
Age, y			
Mean	38.8	46.3	63.2
Median	39.3	46.4	65.7
Days after infection^a^			
Mean	171	314	70
Median	154	252	87

Abbreviation: PCS, post–COVID-19 syndrome.

^a^Time of sampling after initial diagnosis.

### Type I IFN-Specific Autoantibody Measurement in Patient Plasma

For anti-IFN autoantibody detection, a previously described bead-based multiplexed immunoassay with Luminex xMAP technology was adopted [25, 26]. The coupling of the various magnetic microspheres (beads) to the different type I IFN proteins (IFN-α2, Novusbio NBP2-35893; IFN-ß, PeproTech 300-02BC; IFN-ω, Novusbio NBP2-35893) was performed according to manufacturer's instructions.

Prior to patient plasma analysis, all samples were heat inactivated at 56°C for 1 hour. For serologic testing, patient samples were diluted 1:50 in 1% bovine serum albumin (BSA; phosphate-buffered saline [PBS]/BSA) and incubated with equal mixtures of the coated beads (at a concentration of 2000 beads/region/well) for 1 hour at room temperature. A previously used human polyclonal anti-IFN-α2b antiserum served as a positive control (NR-3072; BEI Resources), and an in-house healthy donor pool of human plasma was used as a negative control. After PBS/BSA washing, beads were incubated with a 1:500 dilution of phycoerythrin-labeled goat anti-human IgG secondary antibody (2040-09; Southern Biotech) for 1 hour at room temperature, before being washed twice with PBS/BSA and analyzed on the Luminex FlexMap 3D System. A minimum of 50 beads were analyzed per antigen, from which we determined median fluorescence intensity scores and calculated fold change values over control/empty beads normalized to healthy donor values (ie, fold over empty). Patient plasma exhibiting fold over empty >2 was considered positive.

### Type I IFN Neutralization Activity Measurement

Approximately 2.4 × 10^4^ human embryonic kidney HEK293T cells (CRL-3216; ATCC) were transfected with 30 ng of pGL3-Mx1P-FFluc plasmid encoding firefly luciferase (FF-Luc) under control of the IFN-inducible mouse Mx1 promoter (kindly provided by Georg Kochs) and 4 ng of a constitutively expressed Renilla luciferase (Ren-Luc)–containing plasmid (pRL-TK-Renilla). After incubation for 24 hours, patient plasma samples were diluted 1:50 and incubated for 1 hour at room temperature with 1, 0.2, or 0.04 ng/mL of IFN-β or 10, 1, or 0.2 ng/mL of IFN-ω before addition to the transfected cells. After a further 24 hours, cells were lysed, and FF-Luc and Ren-Luc activity levels were determined with the Dual-Luciferase Reporter Assay System (E1960; Promega) and a PerkinElmer EnVision plate reader (EV2104) according to the manufacturers’ instructions. FF-Luc values were normalized to Ren-Luc values and then to the median luminescence intensity of control wells that had not been stimulated with IFN-β or IFN-ω.

### ISG Signature Measurement

Expression of IFN-stimulated genes (*IFI27*, *IFI44*, *IFI44L*, *IFIT1*, *ISG15*, *RSAD2*, *SIGLEC1*) was measured with quantitative reverse transcriptase polymerase chain reaction in peripheral blood mononuclear cells (PBMCs) and normalized to *GAPDH* and *HPRT1*. Total RNA was extracted from PBMCs via the RNeasy Mini Kit (Qiagen), followed by DNase I digestion. Gene expression was determined with TaqMan Universal PCR Master Mix (Applied Biosystems) on an ABI7300. For calibration, calibrator cDNA was included in each assay. Target genes were analyzed by predesigned TaqMan probes for the IFN-stimulated genes and oligonucleotides targeting *GAPDH* and *HPRT1* as described previously [27]. The IFN score was calculated as previously described [28]. Briefly, the mean and SD levels of each IFN-inducible gene in a normal control were used to standardize expression levels of each gene for each patient. The standardized levels were subsequently summed for each patient to provide an IFN score:


$$\sum_{i=0}^{7}=\frac{Genei\;(PCS)-mean\;Genei\;(Ctrl)}{SD\;(Genei\;(Ctrl))},$$

where *i* = each of the 7 IFN-inducible genes, *Gene i(PCS)* = the gene expression in each patient with PSC, and *Gene i(Ctrl)* = the gene expression in controls.

### Statistical Analysis

Statistical significance was determined with a 2-sided Fisher exact test in Prism software (version 9.0; GraphPad).

## RESULTS

First, we addressed the question of whether autoantibodies to various type I IFNs are more prevalent in plasma derived from patients with PCS (ie, patients who reported new fatigue after SARS-CoV-2 infection) as compared with patients who did not develop fatigue after SARS-CoV-2 infection. In the PCS group, we did not include patients who had severe COVID-19 and reported fatigue for at least 12 weeks after initial diagnosis (mean, 314 days; median, 252 days), as it is known that anti-IFN autoantibodies are relatively prevalent in patients critically ill with COVID-19 (at least 10%) [2, 3].

**Figure 1.**
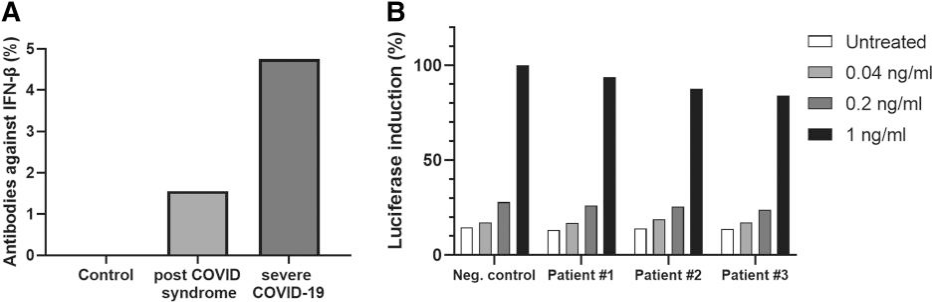
Autoantibodies against IFN-β. *A*, The prevalence of autoantibodies against IFN-β was measured in the plasma of patients: control (n = 38), PCS (n = 128), and severe COVID-19 (n = 21). *B*, The neutralization effect of autoantibodies against IFN-β in plasma from 3 patients with PCS (Nos. 1–3) vs negative control. IFN, interferon; PCS, post–COVID-19 syndrome.

In our PCS cohort with newly developed fatigue, we could not detect autoantibodies against IFN-α2 (Table 2), which is in line with a previous publication [18]. However, 1.56% (2/128) of the analyzed patients with PCS tested positive for binding autoantibodies against IFN-β (Figure 1*A*). Nevertheless, these autoantibodies demonstrated very little neutralizing activity for high levels of IFN-β, indicating that they are unlikely to be clinically relevant (Figure 1*B*). We could not detect autoantibodies against IFN-β in the control group (0/38; SARS-CoV-2 infection, no serious disease, no fatigue), but we did detect them in 1 survivor of severe COVID-19 (1/21, 4.76%). The reactivity was non-neutralizing.

**Table 2.** Prevalence of Autoantibodies Against Type I IFNs

	Control (n = 38)	PCS (n = 128)	Severe COVID-19 (n = 21)
Antibodies against			
IFN-α	0 (0)	0 (0)	0 (0)
IFN-β	0 (0)	2 (1.56)	1 (4.76)
IFN-ω	1 (2.63)	6 (4.69)	3 (14.29)

Data are presented as No. (%).

Abbreviations: IFN, interferon; PCS, post–COVID-19 syndrome.

**Figure 2.**
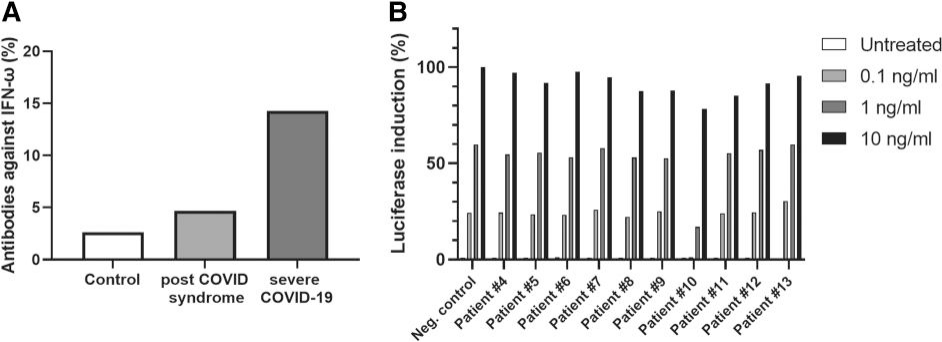
Autoantibodies against IFN-ω. *A*, The prevalence of autoantibodies against IFN-ω was measured in the plasma of patients: control (n = 38), PCS (n = 128), and severe COVID-19 (n = 21). *B*, The neutralization effect of autoantibodies against IFN-ω in the plasma from 10 patients with PCS (Nos. 4–13) vs negative control. IFN, interferon; PCS, post–COVID-19 syndrome.

For IFN-ω, 4.69% (6/128) of the analyzed patients with PCS tested positive for autoantibodies against IFN-ω (Figure 2*A*). However, neutralizing activity could be demonstrated in only a single patient (No. 10; Figure 2*B*). In the control group (SARS-CoV-2 infection, no fatigue, no serious disease), we detected anti-IFN-ω autoantibodies in 1 of 38 (2.63%) individuals, but neutralizing activity was not observed. Notably, 14.29% (3/21) of the analyzed survivors of severe COVID-19 tested positive for autoantibodies against IFN-ω, which is in line with that observed in previous studies (given our low sample number), where 4%–9% prevalence was found in hospitalized patients with severe COVID-19 [3, 25]. Taken together, these findings suggest that autoantibodies against type I IFNs are probably not a relevant factor in PCS, at least in the adult cohort studied here.

**Figure 3.**
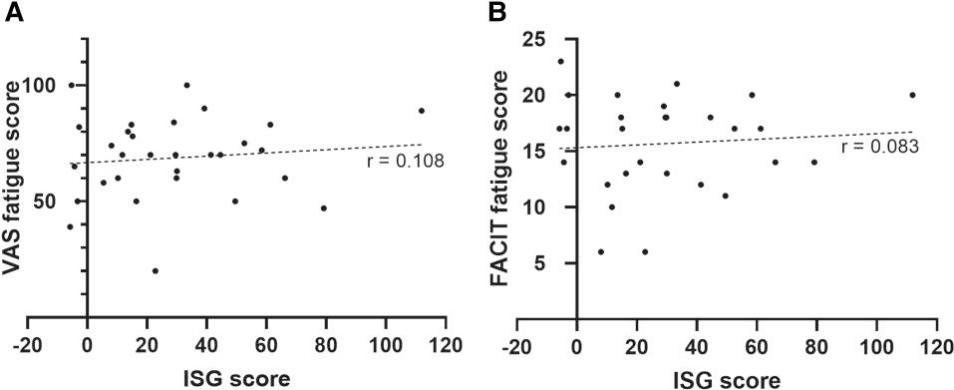
Correlation between ISG signature and fatigue. The ISG signature in a subset of 28 patients with PCS was plotted against the fatigue score measured by (*A*) VAS or (*B*) FACIT-F. Each point shows the ISG value and the fatigue value of an individual patient. The dotted line represents the correlation between the variables. *r* = Pearson correlation coefficient. ISG, interferon-stimulated gene; PCS, post–COVID-19 syndrome; VAS, visual analog scale.

Next, we determined the ISG signature in PBMCs from a subset of our patients with PCS. The ISG score was correlated with the reported fatigue. Fatigue was measured by a visual analog scale and with a questionnaire (FACIT-F) [24]. For both, we could not detect any correlation between the ISG score and the reported fatigue (Figure 3). Altogether, there is no clear evidence that an aberrant IFN system function may be causally related to fatigue in PCS.

## DISCUSSION

In this study, we analyzed the presence of autoantibodies against type I IFNs in a cohort of 128 adult patients with PCS and a control group consisting of 38 asymptomatic patients with a history of mild COVID-19. Contrary to other reports that examined the occurrence of autoantibodies against IFN-α [18], we also analyzed the prevalence of autoantibodies against IFN-β and IFN-ω. The low prevalence of autoantibodies against type I IFNs identified in our study is in line with the previous report that demonstrated the presence of IFN-α2 autoantibodies in around only 2% (2/121) of patients with PCS [18]. While there is a slightly higher prevalence of autoantibodies against type I IFN in our PCS cohort, this is not statistically significant (*P* > .9999 in 2-sided Fisher exact test) and the sample size is small. Consistent with previous observations, our PCS cohort was predominantly composed of females. However, given the prior findings that autoantibodies against type I IFN are predominantly found in men [29], this composition poses a potential risk of underestimating the prevalence of autoantibodies.

Numerous constraints exist in our ability to interpret the outcomes. As shown in Table 1, the control group does not match perfectly to the PCS group. In the PCS group, the mean age is 46 years vs 39 years in the control group. Another relevant bias is the fact that patients of the PCS cohort contacted the clinics because of symptoms and the wish to be treated. The control group consists of volunteers. Moreover, the patients from the PCS cohort were examined, on average, 314 days after infection with SARS-CoV-2, whereas the control group was analyzed 170 days after infection (Table 1). Formally, a comparison between the groups is not correct. Still, in both groups the prevalence of detected autoantibodies against type I IFN was very low. Interestingly, it has also been shown that not all detected autoantibodies effectively neutralize their respective cytokines. In fact, in our study, a potential and clinically relevant neutralizing activity toward IFN-ω was found in only 1 patient, suggesting a low probability of the contribution of autoantibodies against type I IFN to the PCS pathophysiology.

The results of the current study additionally suggest that the prevalence of these autoantibodies in patients with PCS does not substantially differ from their presence in the general population. Autoantibodies against type I IFNs are present in approximately 0.17% to 1.1% of the general population <70 years old [2], indicating that their generation during a lifetime is a rare event and may be associated with acute viral infections, systemic inflammatory events, or chronic type I IFN exposure (eg, in patients with IFN-based anticancer therapy) [6].

Furthermore, a rather lower prevalence of autoantibodies against IFN-β seems to recapitulate findings from patients with critical COVID-19. There, the occurrence of IFN-β autoantibodies is 10-fold lower when compared with autoantibodies targeting IFN-α or IFN-ω. Since the presence of such antibodies might also increase with age, reaching 1.4% to 4.4% in individuals aged ≥70 years [2], their detection in our cohort might have been related to age.

To investigate whether ongoing activation of IFN signaling could be a contributor to PCS pathophysiology, we examined the expression of IFN-stimulated genes (*IFI27*, *IFI44*, *IFI44L*, *IFIT1*, *ISG15*, *RSAD2*, *SIGLEC1*) in peripheral blood mononuclear cells. Next, we calculated individual ISG scores among patients and correlated those results with fatigue perception, which was tested with a visual analog scale and a questionnaire (FACIT-F). However, no correlation was found, suggesting that ongoing activation of the IFN system is not a major driver of PCS. There are several limitations in the interpretation of these results. The IFN score is highly dependent on age, sex, moment of measurement, and interfering infections (unpublished data, M. A. L.-K.).

While an activated IFN system could very well contribute to PCS pathology for single patients, our findings suggest no major universal role of type I IFN dysregulation in adult PCS. However, further studies in larger cohorts that take into account the course of the ISG signature over time are required to better understand the role of the IFN system in patients with PCS.

## Notes

***Author contributions.*** M. A., J. D., N. T., and R. N. R. designed the study. M. A., N. K. M., R. K., N. J., and C. W. performed the experiment, data collection, and data analysis. M.A.P, I. A. A., B. G. H., and S. R. B. supervised the project. W. K., K. d. W., M. A. L.-K., C. S., and S. R. B. acquired funding. M. A., W. K., and C. S. wrote the original draft. M. A., N. K. M., C. S., and B. G. H. reviewed and edited the manuscript. All authors have read and agreed to the published version of the manuscript.

***Financial support*.** This study was supported by the German Research Foundation (project 314061271 to S. R. B., W. K., and C. S. and 288034826 to S. R. B. and C. S.).
